# Demand-pull and environmental innovations: Estimating the effects of innovative public procurement

**DOI:** 10.1016/j.techfore.2017.07.020

**Published:** 2017-12

**Authors:** Claudia Ghisetti

**Affiliations:** European Commission, Joint Research Centre (JRC), Unit I.1, Modelling, Indicators & Impact Evaluation, Via E. Fermi 2749, TP 361, Ispra, VA I-21027, Italy

**Keywords:** Environmental innovation, Government policy, Public procurement, Demand-pull innovation

## Abstract

This paper contributes to the emerging literature on the adoption of environmental innovation, by investigating the so far unexplored role of governmental demand in stimulating ‘greener’ production choices. Specifically, the role of innovative public procurement in driving the adoption and diffusion of sustainable manufacturing technologies is analysed. Results, based on firm-level data in the 28 Member States of the European Union, Switzerland and the USA, are obtained through non-parametric matching techniques. Those outline the crucial role of innovative public procurement in the uptake of environmental innovations. This confirms the relevance of such policy instrument in allowing countries to achieve a decarbonised and sustainable growth path which is compatible with competitiveness goals.

## Highlights

• How public demand affects the adoption of environmental innovations is investigated.
• The public demand instrument scrutinised is “innovative public procurement”.
• The empirical strategy exploits firm level survey data.
• The empirical approach is quasi-experimental based on non-parametric matching.
• Innovative public procurement increases the adoption of environmental innovations.

## 1 Introduction

It is difficult to identify the right amount of resources for the market to invest in knowledge creation. This creates the space for market (Arrow, 1962) or even broader systemic failures. The market may fail to provide adequate levels of research and development (R&D) investments because of the limited appropriability of such activities and the intrinsic uncertainty that characterises any innovation project. This condition may lead to sub-optimal supply of knowledge and, as a consequence, to overall social losses, unless properly designed policies for science, technology and innovation are adopted. Rationales for such policies are discussed by Laranja et al. (2008) and Flanagan et al. (2011).

Within this framework, a broad research effort has aimed to understand the role of specific policies to stimulate innovation. Most of it has been focused on the role of R&D subsidies to counterbalance such under-investment and to stimulate firm's innovative activities, as well as on R&D subsidies' negative side-effects, experienced when they crowd out private investments ((Antonelli and Crespi, 2013, Bloom et al., 2002, David et al., 2000, Hussinger, 2008), among others).

Only recently has there been a turn towards demand-oriented innovation policies, in particular on public procurement (Edler et al., 2012, OECD, 2011), to stimulate innovation, and very few (though robust) empirical analyses have been focused on understanding the effects of public procurement on innovative activities as an alternative or complementary policy instrument (Aschhoff and Sofka, 2009, Guerzoni and Raiteri, 2015). In parallel, a new and fast-growing research field has emerged about a peculiar typology of innovation, that of environmental innovations, whose investigation requires a more systemic lens than ‘standard’ innovations (Rennings, 2000). As these environmental innovations are of importance for both the policy and the business realm and have the potential to lead to win-win solutions whereby competitiveness and environmental sustainability are combined (EEA, 2014), it is relevant to investigate whether or not governmental demand can play a role and foster their development and diffusion.

This article bridges these two research lines and investigates, empirically, whether or not public procurement is a valuable policy instrument to stimulate environmental innovations and, indirectly, to contribute to decoupling economic growth and environmental pressure in order to meet European 2020 and 2030 climate and energy targets (EC, 2014). This is, to the author's knowledge, the first paper to explore such a research question empirically, which is the first way in which this article is original. The analysis of the role of procurement for sustainability is not new; indeed, there are crucial contributions on the topic such as the extensive work of the Organisation for Economic Co-operation and Development (OECD) on sustainable procurement (e.g. (OECD, 2015)) and the United Nations Environmental Programme's work on sustainable procurement (e.g. (UNEP, 2013)). Sustainable procurement has been put at the centre of the international agenda, as the United Nations *2030 Agenda for Sustainable Development* explicitly states the need to ‘promote public procurement practices that are sustainable, in accordance with national policies and priorities’ to reach one of the 17 Sustainable Development Goals (UN, 2015). The main originality lies in the empirical testing of the presence of a statistically relevant effect of procurement in stimulating environmental innovations. The second element of originality is that generalisable results are provided, as the empirical approach is grounded on firm-level data from a wide range of countries: the EU-28, Switzerland and the USA. The empirical approach accounts for the non-randomised nature of the assignment of public procurement tenders to applicant firms by applying a quasi-experimental approach through non-parametric matching techniques.

The rest of the paper is structured as it follows: Section 2 discusses the background literature, Section 3 describes the empirical strategy, Section 4 discusses the main results and Section 5 provides concluding remarks and identifies future lines of research.

## 2 Innovative public procurement and environmental innovations: discussion of the literature

The role of governmental demand in shaping the direction and speed of technological change has been recognised as crucial in the economics of innovation literature: an analysis of seven industries in the USA (semiconductors, commercial aircraft, computers, agriculture, pharmaceuticals, motor vehicles, residential and construction) dating back to 1982 confirmed the pivotal role of public policies in guiding technical progress (Nelson, 1982). Governmental support to innovative activities through public procurement (PP) is seen as a fundamental driver for the uptake of crucial technologies, as happened in the case of general-purpose technologies, which were driven by defence-related procurement in the USA (Ruttan, 2006). Those technologies – mainly steam engines, electric motors and semiconductors – in turn played the role of enabling technologies that fostered widespread technical progress and eventually led to economic growth (Bresnahan and Trajtenberg, 1995). Geroski (1990) expresses a preference for PP over subsidies to stimulate industrial innovation because of subsidies' inefficiencies, characterised by their being ‘unconscionably expensive’ and by the high probability that they attract ‘second-best’ projects in which the rate of return on publicly funded R&D will be lower than that on privately funded R&D. In contrast, Geroski observes that government procurement has a positive net effect on R&D investments over a broad cluster of innovations (including electronic devices, nuclear power, chemical products, and engines and transport equipment). PP is recognised as a successful stimulus for innovation when certain conditions are met: (i) when it expresses a clear and consistent set of needs to be addressed by the innovative effort in a clear contract specification; (ii) when *quality* is placed at the centre of the tender, rather than merely *price*; (iii) when it provides an assured market for early products with uncertain commercial possibilities; and (iv) when it forces contractors to share information and encourages the entry of new competitors so that it stimulates technology diffusion (Geroski, 1990). The author concludes that ‘there is very little question that procurement policy can stimulate industrial innovativeness, and more than a vague suspicion that it can be a far more potent stimulus than a policy of generalised R&D subsidies’ (Geroski, 1990).

Only recently there has been an increasing tendency to reconsider the role of demand-oriented policies in European innovation policies and a discussion has emerged on the role of innovation policies to support ‘Grand Challenges’ in terms of societal and economic goals (Edler et al., 2012, Foray et al., 2012). Those challenges relate to the fields of health, pharmaceuticals, energy, environment, transport and logistics, security, and digital content (Aho et al., 2006). Among the array of demand-side policy instruments, PP helps to reduce the risks linked to innovation investments with unknown demand, very low expected market size or uncertain development, all of which discourage firms from bearing the costs of innovation (Helpman and Trajtenberg, 1994).

In line with this trend, the European Commission has chosen to set a non-binding target of 50% of public tendering to be compliant with its sustainability requirements by 2010, in order to favour improvements in the environmental, energy and social performance of products and services and the development of a Green Public Procurement initiative (EC, 2008). This initiative outlines common criteria to be followed and the need to increase information on the benefits and life cycle costs of environmental friendly products. The strategy has been explicitly linked not only to the creation of market opportunity for existing green small and medium-sized enterprises (SMEs) but also to a stimulus for innovation and diversification in traditional sectors^1^ via the increase in demand for green(er) products and services. In principle, the strategy should stimulate a critical mass of demand for greener goods and services which otherwise would be difficult to get onto the market, as European public authorities are consumers for an amount of EUR 2 trillion per year (16% of the EU's gross domestic product (GDP)) (EC, 2008). Overall, ‘green’ PP is a (procurement) procedure that leads to the purchase of ‘greener’ products, whose impact on the environment throughout their whole life cycle is lower than comparable products or solutions. This provides a stimulus for innovation and creates a minimum critical mass for sustainable goods and services, thus helping to overcome the problem of under-investments in innovation due to the uncertain demand. In reality, this non-binding target has not been reached, as mutually reinforcing obstacles are hindering those organisations that should launch and promote ‘green’ tenders from doing so (for a discussion see (Testa et al., 2016)).

The focus of the current study is not on ‘green’ PP, usually referred to as an environmental policy tool (for a discussion see (Lundberg and Marklund, 2011), or (Parikka-Alhola, 2008)), but rather on *innovative* PP, a category that has recently received attention and is increasingly seen as a crucial instrument for innovation policy. *Regular* PP occurs when a public institution buys already existing products or services for which no R&D is involved and supplier selection depends on readily available information about price, quantity and performance, given the existence of standardised markets (Edquist and Zabala-Iturriagagoitia, 2012, Uyarra and Flanagan, 2010). *Innovative* PP (IPP) occurs whenever public institutions invest in products or services that have not yet been developed but could be developed within a reasonable timeframe, and that can help satisfy human needs or solve societal problems; thus IPP explicitly stimulates an innovative effort (Edquist and Zabala-Iturriagagoitia, 2012). The latter case of procurement (IPP) is the main object of the current study. Public procurement for innovation has been acknowledged as an important demand-side policy instrument, as it has ‘the potential to improve delivery of public policy and services, often generating improved innovative dynamics and benefits from the associated *spillovers*’, but, at the same time, ‘it has been neglected or downplayed for many years’ (Edler and Georghiou, 2007), probably because of the stringent competition rules adopted in Europe (Edquist et al., 2000).

The rationale for using PP to stimulate innovation is threefold, as discussed by Edler and Georghiou (2007): (i) IPP is a major part of local demand and this affects decisions by multinational enterprises (MNEs) about where to locate and the dynamics of innovation in countries; (ii) IPP can help overcome market (information asymmetries) and system (poor interaction) failures relating to innovative products; and (iii) purchasing innovative solutions contributes to improving public infrastructure and services. Intelligent and tailored intermediation services may, however, be needed to make this instrument more effective in connecting supply and demand (Edler and Yeow, 2016). Predicting longer term societal needs and trends in emerging technologies can also make this instrument more effective, as discussed in the case of an Italian regional innovation policy by Vecchiato and Roveda (2014). Overall, IPP is an important source of innovation because it counteracts market and systemic innovation failures, which otherwise would lead to under-investment in innovative activity (Edler and Georghiou, 2007).

Empirical analyses of the role of IPP in stimulating innovation are surprisingly rather scarce, but they strongly confirm that it is a driver of innovation (Aschhoff and Sofka, 2009, Guerzoni and Raiteri, 2015). Aschhoff and Sofka (2009) focus on German firms and find that PP leads to heterogeneous effects on firms' innovation performance: it is particularly effective for smaller firms in geographical areas under economic stress, thus suggesting that it may be of particular relevance for firms facing limited resource constraints. Guerzoni and Raiteri (2015) provide original evidence on how the interactions of demand- and supply-side technology policies affect firms' innovative activities, finding that the interaction of R&D subsidies, R&D tax credits and IPP helps explain innovation, but also that IPP is more effective than R&D subsidies in stimulating innovation.

This theoretical and empirical literature is bridged by an emerging research field on a peculiar subset of innovations, environmental innovations (EI), which are characterised by special features (Rennings, 2000) and a multifaceted nature, which place their investigation at the crossroads between environmental economics and innovation studies (Ghisetti et al., 2015, Horbach et al., 2012, Horbach et al., 2013).

Academic contributions to this emerging research field (for a review see (Barbieri et al., 2016)) acknowledge that environmental regulation and standards provide valuable incentives for EI uptake and diffusion, although some articles find statistically significant results and others do not (Ghisetti and Pontoni, 2015). Furthermore, by analysing European SMEs and the barriers to their innovative activities, Marin et al. (2015) identify six clusters of heterogeneous ‘eco-innovators’ profiles; they stress that this diversity has to be accounted for when designing environmental and innovation policies, as policies can have different effects on firms in different clusters. From a theoretical point of view, governments can stimulate EI by exploiting multiple (even combined) instruments: (i) environmental policy instruments, such as market-/incentive-based instruments, command and control regulation instruments, voluntary agreements or information-/education-based instruments; and/or (ii) innovation policy instruments, such as enforcing/easing the intellectual property right (IPR) protection mechanism, allocating tax credits for innovation, subsidising R&D activities, favouring public R&D or establishing technology transfer instruments (Del Rìo et al., 2010).

Recently it has been argued that PP can be useful to support public research to facilitate advances at the technological frontier and also to meet the EU 2020 targets of socio-economic and environmental sustainability. The uptake of climate-friendly technologies (namely EI) may be influenced by public policies, since the transition to more sustainable production requires the invention, adoption and diffusion of radical and, consequently, riskier innovations, and high levels of investment, which are unlikely to be sustainable by the private sector on its own (Mowery et al., 2010). Alternative-energy technologies are potentially deployed to replace already existing technologies and, initially, may be less reliable and/or more costly, and require public support for their early-stage deployment. As IPP reduces the investment risks inherent in radical innovations with uncertain markets and demand, and it can create niches for the emergence of early-stage environmental technologies, it can be a valuable instrument to encourage their early adoption and to allow their easier diffusion. Furthermore, the more radical the innovation, the higher the entry and switching costs; the lack of adequate policy instruments can be associated with technological lock-in and path dependency effects favouring ‘dirtier’ established technologies (Oltra and Saint Jean, 2009). As IPP can create niches that EI can exploit, coherently with the ‘lead market approach’ (Beise and Rennings, 2005, Edler and Georghiou, 2007, Horbach et al., 2014), it can favour the early-stage adoption of EI to make them widespread afterwards. Overall, there is agreement that governmental intervention is needed to favour the adoption and diffusion of EI and that IPP might be well positioned to be a valuable policy instrument to this end. However, there is still no empirical confirmation that IPP effectively stimulates EI.

This paper draws on the literature described so far and it fills this research gap by empirically testing whether or not IPP affects firms' environmental strategies. As stated in the introduction, it is the first empirical analysis testing the role of IPP as a policy instrument for EI. For the reasons outlined above, the expectation, and consequently the main research hypothesis, is that IPP positively influences firms' choices towards the adoption of EI.

## 3 Data and empirical strategy

The recent Innobarometer dataset, entitled *Flash Eurobarometer 415*: *Innobarometer 2015* - *The Innovation Trends at EU Enterprises* (EC, 2015), is exploited for the empirical strategy. The survey was carried out in February 2015 by the consortium TNS Political and Social using a computer-assisted telephone interviewing (CATI) system, at the request of the European Commission Directorate-General for Internal Market, Industry, Entrepreneurship and SMEs (DG GROW) and under the coordination of the Directorate-General for Communication. It covers businesses with one or more employees in the manufacturing, service and industry sectors and the sample is stratified by size, sector and country. Respondents were general managers, financial directors or owners. It is a valuable source of information, as it is the only available survey that combines, at the firm level, information on public procurement with information on the adoption of environmental innovations. Data on the role of IPP and the adoption of sustainable manufacturing technologies (or EI) are collected only for manufacturing firms; therefore, the analysis in this paper focuses on manufacturing firms in the EU, Switzerland and the USA and covers all businesses with one or more employees. The full sample, representative of all sectors, amounts to 14,118, but only 3018 of them are manufacturing firms, thus limiting the operative sample to the latter number, which falls to 3001 after cleaning for the missing variables of interest.

The dependent variable EI takes the value 1 whether the firm has adopted sustainable manufacturing technologies or is planning to adopt them in the next 12 months. Those are defined in the survey as technologies which use energy and materials more efficiently and drastically reduce emissions.

The core variable of interest, IPP, exploits two questions of the survey, the first aimed at scrutinising whether or not the firm has participated (with or without success) in a PP tender and the second aimed at understanding whether or not innovation was part of the contract (see Table 2 for the full text of the questions). Unfortunately, no information is available on how firms submit their tender proposals and no qualitative information on the tender is provided.^2^ An overview of the responses given to the first of these questions gives an interesting and qualitative picture on the nature and magnitude of the PP phenomenon. In the operative sample, 30% of respondents (915 firms) declared that they had submitted at least one tender for a PP contract since January 2012 (with a positive, unknown or negative outcome) and 24% of respondents (702 firms) had won at least one PP tender. Interestingly enough, 44% of respondents that had won at least a PP tender declared that they had included innovations as part of the procurement contract.

To construct the empirical strategy properly, the main empirical issue to consider is that the assignment of IPP to firms may be non-randomised: (i) firms submitting a IPP proposal may be self-selected on certain observable characteristics; or (ii) public agencies may try to maximise the effects of the policy by imposing selection criteria related to firms' characteristics, as part of a strategy of ‘picking the winner’ or (contrarily) ‘aiding the poor’. In other words, there is a potential selection problem both on the side of firms and on the side of public funding agencies. Estimating the effect of IPP on EI directly in such a setting could distort the interpretation of results, since the magnitude of the difference in environmental innovativeness depends not only on the consequences of the technology policy.^3^ The non-neutral application and funding process, thoroughly discussed by David et al. (2000), suggests the need to rule out problems of simultaneity and selection bias and to choose a quasi-experimental approach and non-parametric matching method, widely applied in public funding evaluation articles in the field of industrial economics (Almus and Czarnitzki, 2003, Czarnitzki and Lopes-Bento, 2014).

This approach considers IPP to be the treatment variable and divides the total sample (3001 firms) into a treated group (308 firms) and a control group (2693 firms), depending on whether or not firms won a tender for a PP contract explicitly requiring innovations. As expected, and confirmed by the statistics reported in Table 1, the two groups are significantly different. Treated firms are more likely to be environmental innovators, are older, are more innovative and have fewer employees than control firms, as is confirmed by *t*-tests comparing mean differences, whose significance is reported in the last column.

**Table 1:** Description of the variables, statistics and *t*-test on mean differences.

Variable	Description	Min.	Max.	Mean			
				Full sample (*n* = 3001)	Treated (IPP = 1)	Control (IPP = 0)	*t*-Test on mean differences
EI	The company has adopted sustainable manufacturing technologies or plans to adopt them in the next 12 months	0	1	0.381	0.497	0.368	⁎
FIN	The company perceives a lack of financial resources	0	1	0.251	0.221	0.255	NS
GROUP	The company is part of a group	0	1	0.313	0.351	0.308	NS
Lsize	Natural logarithm of full-time equivalent employees	0	10.08	3.478	3.84	3.44	⁎
IPP	The company won at least one PP contract and included an innovation as part of this contract	0	1	0.103	–	–	–
RD	The company invests in R&D activities	0	1	0.528	0.660	0.513	⁎
Size	Firm's size measured by full-time equivalent employees	1	23,764	162.78	289.16	148.33	⁎
YOUNG	The company was established after January 2014	0	1	0.0746	0.045	0.778	⁎

⁎ Variable mean differences between the two groups are statistically different from zero (*t*-test *p*-value < 0.05); NS, the difference between the groups is not statistically different.

The average treatment effect on the treated firms (ATT), that is the estimation of the effects of IPP on the outcome (EI), is equal to the difference between the average of the target variable EI when the firm is treated (Y_1_) and the average of the target variable EI when the same firm is untreated (Y_0_).

Under random assignment, the independence assumption in Eq. (1) holds, IPP is fully exogenous and the ATT effects of the treatment (IPP) could be estimated as simple between-groups mean difference in the outcome variable EI, as in Eq. (2).

(1) $$\left(Y_{1};Y_{0}\right)⊥\mathrm{IPP}$$

(2) $$E\left(Y|\mathrm{IPP}=1\right)-E\left(Y|\mathrm{IPP}=0\right)=E\left(Y_{1}|\mathrm{IPP}=1\right)-E\left(Y_{0}|\mathrm{IPP}=0\right)=E\left(Y_{1}\right)-E\left(Y_{0}\right)=\mathrm{ATT}\left(=\mathrm{ATE}\right)$$

However, as explicitly discussed in this section, the independence assumption does not hold in this setting, as IPP is not exogenous; rather, it is expected to be influenced by certain observable characteristics of firms that drive selection of who gets the treatment and who is excluded.

To properly estimate the effect of IPP as the difference in EI outcomes depending only on it, ATT should be estimated as in Eq. (3), and each treated firm needs to be compared with a hypothetical scenario where the same firm receives no treatment, as the treated and control groups would be different in the outcome even in the absence of the treatment, or, formally:

(3) $$\mathrm{ATT}=E\left[Y_{1}-Y_{0}|\mathrm{IPP}=1\right]+E\left[Y_{0}|\mathrm{IPP}=1\right]-E\left[Y_{0}|\mathrm{IPP}=0\right]$$

where:

(4) $$E\left[Y_{0}|\mathrm{IPP}=1\right]-E\left[Y_{0}|\mathrm{IPP}=0\right]\neq 0$$

Since the counterfactual situation E[Y_0_ | IPP = 1] is not observable, as it is not possible to forecast a recipient firm's EI performance in the absence of the treatment, a non-parametric approach is applied to create comparable groups of firms. Specifically, propensity score matching (PSM) is exploited to create the best pairs of treated and control firms. This matching is needed to be able to compare the means of outcomes of paired firms in EI in order to estimate ultimately the unbiased effect of the public funding IPP. This pairing is conducted on the basis of their propensity score values, a methodology that allows us to summarise the multidimensionality of the matching criteria as one single value, the propensity score, aimed at capturing the probability that firm *i* accesses the treatment (Rosenbaum and Rubin, 1983). If the common support condition, the stable unit treatment value assumption and the unconfoundedness, or conditional independence assumption, hold, the average treatment effect can be estimated as the difference in the outcome variable EI between the two paired groups of firms, namely treated and control firms. The stable unit treatment value assumption (SUTVA) assumes that the treatment of firm *i* does not affect the outcome of another unit. The common support condition requires that the vector of chosen covariates does not perfectly predict the outcome: covariates themselves should not perfectly predict whether firm *i* receives PP or not. The conditional independence assumption requires that, once it is controlled for all *observable* variables, the potential outcomes are independent of treatment assignment. In this framework, no unobserved factor should influence both PP and EI. Cerulli (2015) discusses the implications and tests of these conditions. Should all these conditions hold, PSM produces unbiased estimates of the effect of the treatment (IPP) on the outcome (EI).

Propensity scores are computed as the probability of receiving the treatment conditional on a set of observable characteristics that simultaneously influence the decision to engage in IPP and the outcome EI. Selected observable characteristics, constructed at the firm level, are the perception of financial constraints as a barrier to firms' innovative activity (FIN), being part of a group (GROUP), the size of the firm in terms of full-time equivalent employees, as a natural logarithm (Lsize), a positive investment in R&D activities (RD), whether the firm is young or not, depending whether it was established after or before January 2014 (YOUNG), and seven sector dummies.^4^ Table 2 reports the questions asked in the survey and links them to the aforementioned variables.

**Table 2:** Questions asked in the survey.

Question	Variable
• (Q11A) Have you used any of the following technologies? (Y/N) ◦ Sustainable manufacturing technologies (i.e. technologies which use energy and materials more efficiently and drastically reduce emissions) • (Q11B) Do you plan to use any of the following technologies in the next 12 months? ◦ Sustainable manufacturing technologies (i.e. technologies which use energy and materials more efficiently and drastically reduce emissions)	EI
• (Q12) Since January 2012 has your company (One possible choice): ◦ Won at least one public procurement^a^ contract ◦ Submitted at least one tender for a public procurement contract and the outcome is unknown ◦ Submitted at least one tender for a public procurement contract without success ◦ Investigated opportunities to bid on one or more public procurement contracts, but have never submitted a tender ◦ Has never submitted a tender nor investigated opportunities to bid on a public procurement contract • (Q13) Has your company included any of its innovations as part of any public procurement contract that you have won? (Y/N)	IPP
• (D2) When was your company established (One possible choice): ◦ Before 1 January 2009 ◦ Between 1 January 2009 and 1 January 2014 ◦ After 1 January 2014	YOUNG
• (D4) Is your company part of a group? (Y/N)	GROUP
• (Q5A–Q5B) Thinking about the commercialisation of your company's innovative goods or services since January 2012, have any of the following been a major problem, a minor problem or not a problem at all? ◦ Lack of financial resources: a major problem	FIN
• (Q4_4) Since January 2012, what percentage of its total turnover has your company invested in Research and development (R&D)? ◦ 0% ◦ < 1% ◦ 1–5% ◦ > 5%	RD
• Size of the company, sample information	Lsize

Note: Additional possible answers to the original questions which are not relevant have been omitted to improve the clarity of the table.

a The read-out provided to the respondents states: ‘the term “public procurement” describes the purchase of goods, services and public works by governments and public bodies’.

**Fig. 1:**
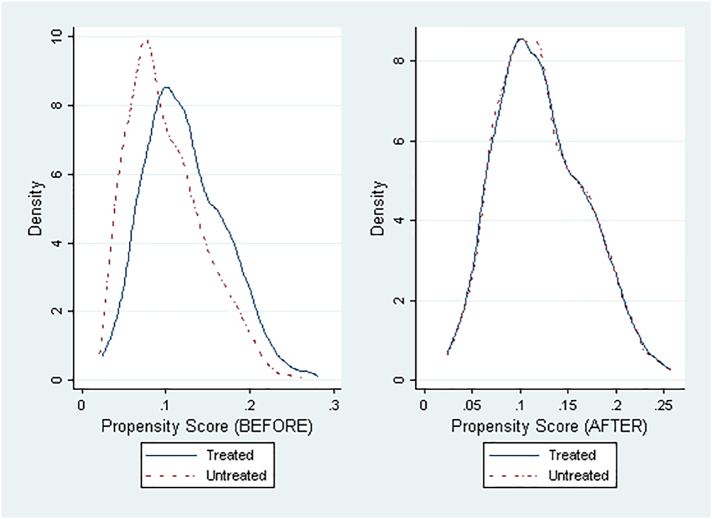
Propensity score distribution for treated and control groups before and after matching.

Fig. 1 depicts the propensity score distribution between the groups, before and after the matching, and shows the reduction in group differences after the matching and its good quality.

**Fig. 2:**
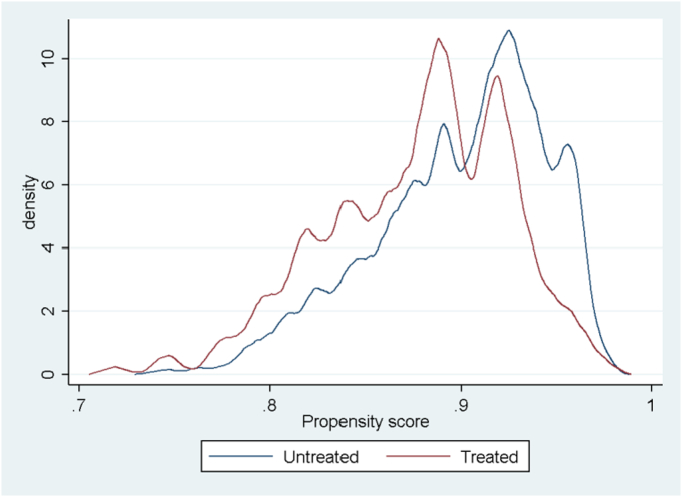
Overlap assumption.

Fig. 2 depicts the test for the overlap assumption and shows there is no evidence of this being violated: there is no high probability mass close to 0 or 1, and there are two estimated densities with most of their masses in regions where they overlap.

## 4 Discussion of results

Once the quality of the propensity scores is confirmed, they are used for the non-parametric matching. The choice is to ground the matching on alternative algorithms to better balance the trade-off between bias and efficiency (Caliendo and Kopeinig, 2008). The three nearest neighbour matching (3NNM) algorithm is the selected algorithm; to provide robustness, results under alternative algorithm are also reported. The 3NNM method selects for comparison only the three observations whose propensity scores values are closest to those of treated firm *i*. The alternative algorithms presented are a single nearest neighbour matching (NNM), a 3NNM with a *caliper* set to impose a minimum degree of quality on the matching, a five nearest neighbour matching (5NNM) and a kernel matching estimator with bootstrapped standard errors (1000 repetitions).^5^ All tests for matching quality support the validity of the matching: regression *t*-tests on differences in covariates means after the matching shows that all *p*-values are lower than 0.05, the log-likelihood ratio is rejected before the matching and not rejected after the matching, pseudo-*R*^2^ is lower in the matched than in the unmatched firms and the standardised mean bias test falls below 5%.

The results of the 3NNM on NNM, 3NNM with a *caliper*, 5NNM and kernel matching are reported in Table 3, column (1).

**Table 3:** Results and robustness of alternative algorithm and alternative sampling.

Algorithm	(1)	(2)	(3)
	ATT	ATT reduced sample	ATT outcome INNO
**3NNM**	**0.111**⁎⁎⁎	0.112⁎⁎⁎	0.066⁎⁎⁎
	(0.033)	(0.037)	(0.025)
NNM	0122⁎⁎⁎	0.101⁎⁎⁎	0.067⁎⁎
	(0.038)	(0.041)	(0.028)
3NNM caliper^a^	0.098⁎⁎⁎	0.150⁎⁎⁎	0.064⁎⁎
	(0.034)	(0.037)	(0.025)
5NNM	0.107⁎⁎⁎	0.116⁎⁎⁎	0.060⁎⁎
	(0.032)	(0.036)	(0.024)
Kernel^b^	0.121⁎⁎⁎	0.126⁎⁎⁎	0.077⁎⁎⁎
	(0.029)	(0.032)	(0.024)

Note: Standard error in parentheses. Preferred algorithm in bold.

^⁎^*p* < 0.10.

⁎⁎ *p* < 0.05.

⁎⁎⁎ *p* < 0.01.

a Caliper = 0.25 times propensity scores' standard error. It equals 0.0114 in the whole sample (columns (1) and (3)) and 0.0198 in the reduced sample (column (2)).

b Bootstrapped standard error, 1000 repetitions.

Overall, IPP is a significant driver of EI: the ATT (that is, the difference in outcome averages between the treated and control groups after pairing) is positive and significant for the alternative matching algorithm adopted. The number of firms that are environmentally innovative after being treated by IPP is 11.1 percentage points higher in the treated group than in the control group. This result is comparable across the alternative matching algorithms selected.

To better control for the validity of the results, an important sensitivity test is conducted and reported in Table 3, column (2). The homogeneity of firms in the two groups (treated and control) is increased by excluding firms that said they had never submitted an IPP tender or explored the possibility of doing so. The rationale for this sensitivity test is that control and treated group might be too different to be comparable, even after matching, when the first includes firm that have not even tried to participate in a tender, in case those are structurally different from firms that have tried (successfully or not) to win a PP tender. This poses the risk of affecting the estimation of the effect of IPP for treated firms. After this exclusion, the treated group remains unaltered (308 firms) while the control group is reduced to 945 firms. The quality of the matching is confirmed and is reported in the Appendix A. Even with the exclusion, results, reported in column (2), largely confirm previous findings. Firms that are environmentally innovative after treatment by IPP are 11.2 percentage points higher in the treated group than in the control group.

Finally, to provide a more comprehensive picture of IPP, its effects on more ‘standard’ innovative outcome are estimated. A variable INNO is constructed, which is equal to 1 if firms are either product or process innovators, and it is used as an outcome variable in the estimates of ATT of IPP treatment. Results (column (3)) show that the effect on more standard innovative outcomes remains significant and positive but is half of that for EI: the number of firms in the treated group is only 6.6 percentage points higher than in the control group. In relation to EI, IPP has twice the effect of standard innovations.

This interesting result can be explained by the introduction of specific policy commitments towards ‘green’ PP, such as the European Green Public Procurement Initiative (EC, 2008). Those explicit policy commitments towards a green-oriented PP may have led to a transition towards greener PP tenders and, as a consequence, to a higher increase in the environmental innovativeness of firms than in their ‘standard’ innovativeness.^6^

All in all, the results largely confirm that this policy instrument has a positive and significant effect and highlight the need to consider it to build a policy mix to stimulate firms' environmental innovativeness and, more broadly, the transition to a more sustainable society.

An explanation of these results, and in particular through which mechanisms IPP affects EI, comes from the literature reviewed in Section 2. As the full social costs of greenhouse gas emissions and pollution in general are not (yet) reflected in market prices (Fischer and Newell, 2008, Newell, 2010), there is room for public policies to compensate for the risk of under-investment in environmental innovations due to the absence of proper market signalling. Furthermore, the early versions of most alternative energy technologies would be handicapped in comparison with existing, dirtier technologies from the point of view of prospective adopters, given the high risks associated with those technologies in all stages of the innovation process, from invention to development to commercialisation and diffusion. EI may thus require public support, particularly for early adopters of those technologies. Given the features of PP as an instrument that helps to reduce the risks of innovation investments for which the demand is unknown, the expected market size is very low or development is uncertain, the paper argues that PP may be well placed to stimulate EI, as they may suffer (even more than ‘standard’ innovation) from technological lock-in in favour of dirtier and more established technologies.

Before we discuss the main policy implications of these findings, the next subsection outlines an additional robustness control that has been performed to confirm the validity of the results.

### 4.1 Robustness checks on environmental regulation

As a further robustness control, the role of existing environmental regulation in spurring the adoption of EI is accounted for. As the literature on EI largely agrees that regulatory push and pull stimulus is a core determinant of EI (Frondel et al., 2008, Ghisetti and Pontoni, 2015, Horbach et al., 2012, Rennings, 2000), its omission may bias the results, if EI adoption is driven by specific environmental regulation rather than by any IPP tender. The OECD Environmental Policy Stringency composite indicator (OECD EPS) is used to extract country-level indices on the stringency of environmental regulation. This index, ranging from 0 to 6, is selected because it allows international comparability over time (Botta and Koźluk, 2014). It measures the stringency of environmental regulation by putting an explicit or implicit price on polluting or environmentally harmful behaviours and it is based on the degree of stringency of multiple environmental policy instruments, primarily related to climate and air pollution.

As this index is not available for the whole set of countries covered in the previous sections, this analysis is considered only as a robustness control rather than being the principal choice. The following countries are excluded from the robustness check because of data limitations: Bulgaria, Croatia, Cyprus, Estonia, Latvia, Lithuania, Luxembourg, Malta, Romania and Slovenia. The final number of observations falls to 2238, of which 235 are treated and 2003 are not. The last available year (2012) of the index is used to construct the variable EPS, which has been included as an additional explanatory variable of EI.

The results, reported in Table 4, are consistent with previously outlined evidence: ATT using a 3NNM is significant and equals 0.121; it equals 0.129 when a *caliper* of 0.0125 is imposed.

**Table 4:** Results and robustness of alternative algorithm and alternative sampling.

Algorithm	ATT including OECD EPS variable
	(*N* = 2238)
**3NNM**	**0.121**⁎⁎⁎
	(0.0404)
3NNM caliper of 0.0125	0.129⁎⁎⁎
	(0.0395)

Standard error in parentheses. Preferred algorithm in bold.

⁎⁎⁎ *p* < 0.01.

The previous findings – although not directly comparable, given that *n* changes, as do the included countries – are confirmed: the number of firms that are environmentally innovative after treatment by IPP is 12 percentage points higher in the treated group than in the control group, even after controlling for environmental regulation.

## 5 Concluding remarks

The results previously discussed confirm the expectation that IPP has a role in stimulating the uptake of EI, and thus, indirectly, in contributing to the grand societal challenge of climate change. This evidence has been proven to be robust to different robustness controls.

Procurement is increasingly seen as a way to improve sustainability. The United Nations 2030 Agenda for Sustainable Development explicitly refers to the need for countries to promote *sustainable procurement* as one of the Sustainable Development Goals (UN, 2015). The European Commission's Green Public Procurement initiative (EC, 2008) sets a non-binding green public procurement target to favour improvements in the environmental, energy and social performance of products and services and to stimulate their development. The present paper has combined this view in the current article with the increasing interest in procurement as a way to stimulate innovation, as pointed out in the discussion of the innovation literature in Section 2.

All in all, the current empirical findings allow us to add a piece of information that is relevant to shaping environmental as well as innovation policies, with important policy implications. Overall, they support the view that IPP can play a role in improving sustainability through the increased adoption of EI by firms. This would allow the discussion on the role of sustainable procurement in reaching sustainability targets to merge with that on the role of innovative procurement in stimulating innovation, so that we can reach the final synthesis that IPP is a policy tool that can positively stimulate not only (standard) innovations but also the peculiar typology of EI. The latter are capable of hitting environmental as well as economic sustainability targets, possibly leading to win-win outcomes (EEA, 2014) and helping the transition to a more sustainable society.

The results of this article go in this direction and suggest that demand can affect the rate of adoption of EI and, more precisely, that IPP strongly stimulates EI adoption. This would call for including IPP in the array of innovation policy instruments as well as in the array of regulatory push-pull instruments for decarbonisation, to allow us to meet the sustainability targets that have been set. The literature has indeed already confirmed the role of EI in enhancing competitiveness (Ambec et al., 2008, Porter and Van der Linde, 1995) and reducing environmental pressure (Ghisetti and Quatraro, 2017). The diverse nature of innovations, and EI more specifically, indicates the need to be cautious, as a one-size-fits-all model of procurement is unlikely to work: as discussed by Uyarra and Flanagan (Uyarra and Flanagan, 2010), the innovative procurement model might not work in all procurement contexts and for all types of goods and services.

A limitation of the current study is that the data used do not shed light on the design of the PP instrument, which instead affects how successful this instrument is in stimulating innovation (Geroski, 1990). PP policy measures might suffer from the presence of perceived barriers to the suppliers (Uyarra et al., 2014) and from the lack of a systematic basis in their design, which results in deficiencies that eventually cause the policy to fail (Georghiou et al., 2014). A further limitation is the cross-sectional nature of the data, which does not allow the formulation of causal connections between the variables analysed.

The current research has implications for future studies. It has identified interesting research directions, and left them to future research because of constraints and limitations on the data. Quantitative information on the number of tenders that each firm applied for or (even better) won is not available, and it would be of interest to test if IPP affects EI differently depending on whether the firm has a broad and/or deep experience in tenders or is rather immature in this field. Secondly, it would be an interesting extension of this work to consider the role of a policy mix that includes IPP policy tools in stimulating EI, as Guerzoni and Raiteri (2015) do with respect to ‘standard’ innovations. The last interesting extension would be to use panel data to improve the conclusion about whether or not there are causal connections between EI and IPP.

## Figures and Tables

**Fig. A1:**
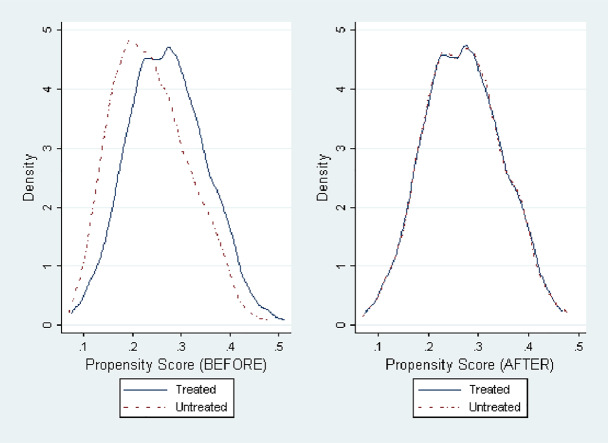
Distribution of propensity scores in reduced sample.

**Fig. A2:**
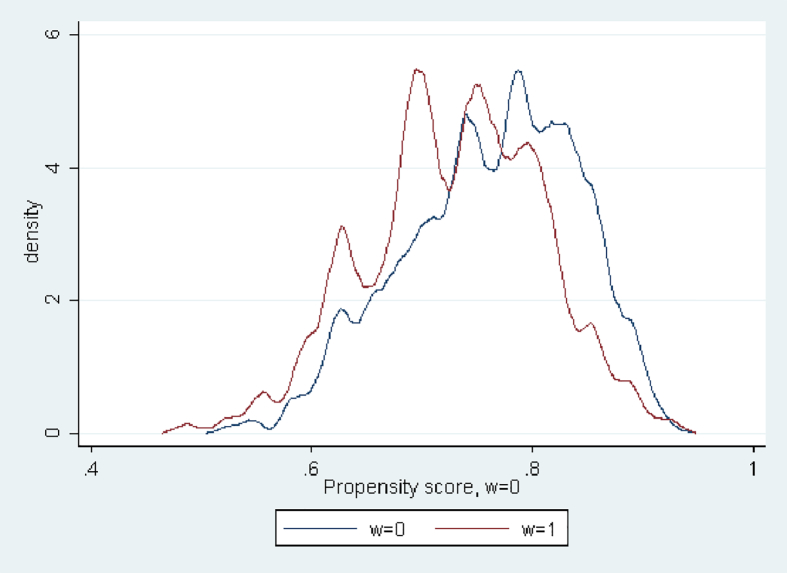
Overlap assumption in reduced sample.

**Table A1:** Robustness on alternative algorithm on subsample of firms.

	Propensity score	Propensity score on reduced sample
Lsize	0.1156⁎⁎⁎	0.1090⁎⁎
	(0.0425)	(0.0455)
GROUP	− 0.1518	− 0.1690
	(0.1529)	(0.1633)
YOUNG	− 0.4084	− 0.3905
	(0.2856)	(0.3055)
RD	0.4835⁎⁎⁎	0.4171⁎⁎⁎
	(0.1323)	(0.1410)
FIN	− 0.0546	− 0.2112
	(0.1495)	(0.1618)
Constant	− 2.4237⁎⁎⁎	− 1.5670⁎⁎⁎
	(0.2088)	(0.2289)
*N*	3001	1253
Pseudo-*R*^2^	0.0336	0.0307

Note: Standard error in parentheses. Seven sector dummies included.

^⁎^*p* < 0.10.

⁎⁎ *p* < 0.05.

⁎⁎⁎ *p* < 0.01.

## Appendix A

Estimates of the treatment effects of IPP have been carried out on a smaller sample of firms in the control group to exclude all firms that said they had never submitted a PP tender or explored this possibility. This would in principle increase the homogeneity of firms between the treated and control groups to compare firms that have won a PP tender and those that have at least tried to win one. The methodology is the same described in the article; the chosen matching algorithm is the 3NNM. Tests confirm the quality of the matching (Fig. A1, Fig. A2).

Results of the first step logistic estimation of the propensity scores used for the matchings in the article are reported in Table A1. The first column shows those of the full sample and the second column shows those of the reduced sample.

## Footnotes

1 Although the traditional sector might not benefit from green procurement stimulus because it lacks systemic perspectives and skills in negotiation and inter-organisational planning (Rizzi et al., 2014).

2 Limitations of this point are discussed in the concluding section.

3 A logit model is estimated with EI as the dependent variable and IPP, standard control variables, and state and sector dummies as the explanatory variables, and a significant positive effect of PP on EI is found. However, this result (estimates available upon request) is not reliable given the sources of bias described. Accordingly, this result will not be presented.

4 The seven sector dummies included are CA, CB, CC, CD-CG, CH, CI-CL and CM (Statistical classification of economic activities in the European Community (NACE) Second Revision). Because of data constraints it was not possible to impose country as an observable characteristic upon which to construct the pairing of treated and control firms, as none of the algorithms succeeded in finding, within each and every country (30 in the sample), pairs of firms which were similar enough in terms of the remaining observable characteristics.

5 The *teffects psmatch* command in Stata14 is used to calculate ATT, since it estimates standard errors adjusted for the first-step estimation of propensity scores, as suggested by Abadie and Imbens (2016). The balancing property of the propensity score is tested using the Becker and Ichino (2002) user-written Stata command pscore and is satisfied.

6 Unfortunately the dataset allows us only to infer this interpretation, as it contains no information on whether or not the IPP tender also included environmental requirements. Consequently, it cannot be properly tested and it is left for future research.
